# Redox Status of *β*_2_GPI in Different Stages of Diabetic Angiopathy

**DOI:** 10.1155/2016/8246839

**Published:** 2016-10-13

**Authors:** Jun Ma, Jing-Yun Zhang, Yan Liu, De-Min Yu, Pei Yu

**Affiliations:** ^1^Key Laboratory of Hormones and Development (Ministry of Health), Tianjin Key Laboratory of Metabolic Diseases, Tianjin Metabolic Diseases Hospital & Tianjin Institute of Endocrinology, Tianjin Medical University, Tianjin 300070, China; ^2^Tianjin Haibin People's Hospital, Tianjin, China

## Abstract

We explored the redox status of beta 2 glycoprotein I (*β*_2_GPI) in different stages of diabetic angiopathy. Type 2 diabetes mellitus (T2DM) had a significantly lower proportion of reduced *β*_2_GPI as compared to healthy controls (*p* < 0.05). There was a trend that the mild coronal atherosclerosis heart disease (CAD) had higher proportion of reduced *β*_2_GPI than non-CAD and severe-CAD groups, however without significances (*p* > 0.05). The mild-A-stenosis group and mild-diabetic retinopathy (DR) groups had higher proportion of reduced *β*_2_GPI than their severely affected counterparts. The mild-slow nerve conduction velocity (NCVS) group had higher proportion of reduced *β*_2_GPI than normal nerve conduction velocity (NCVN group) and severe-NCVS groups. The proportion of reduced *β*_2_GPI was in positive correlation with 24 h urine microalbumin and total urine protein, and the proportion of reduced *β*_2_GPI was in negative correlation with serum and skin advanced glycation end products (AGEs). Taken together, our data implicate that the proportion of reduced *β*_2_GPI increased in the early stage of angiopathy and decreased with the aggravation of angiopathy.

## 1. Introduction

Cardiovascular disease (CVD), blindness, renal failure, and amputation caused by diabetic angiopathy contribute to healthy burden in modern society. The pathological mechanism of diabetic angiopathy is still unclear, although oxidative stress (ROS) is one of the critical initiating factors in diabetic complications [1].

Beta 2 glycoprotein I (*β*_2_GPI) is a type of single-strand protein that contains five structural domains (DI-DV). As the major autoantigen of antiphospholipid syndrome (APS), *β*_2_GPI is closely associated with thrombotic events in patients with APS [2]. The Cys288 to Cys326 disulfide bond in domain V can be reduced by oxidoreductase enzymes thioredoxin (TRX-1) and protein disulfide isomerase (PDI). Free thiol-containing *β*_2_GPI was discovered in 2010 [3] and this reduced *β*_2_GPI, in contrast to oxidized *β*_2_GPI, can protect endothelial cells from oxidative stress [4]. Research shows that the APS patients have significantly higher serum oxidized *β*_2_GPI level than that in healthy controls, and reduced *β*_2_GPI level is significantly reduced in APS patients. Our previous work has shown that reduced *β*_2_GPI could inhibit the formation of foam cells and macrophage apoptosis [5], protect endothelial cells from oxidative stress-induced cell injury [4], inhibit retinal angiogenesis in diabetic rats, and reduce expression of collagen type IV in diabetic kidney. Conversely, an imbalanced redox state of *β*_2_GPI may be important to increase thrombotic events in APS, and *β*_2_GPI levels could form the basis of thrombosis risk [2].

Diabetics tend to be exposed to high oxidative stress state due to elevated radical ROS production caused by increased advanced glycation end products (AGEs), polyol pathway, and other factors. However, there is no report regarding the redox balance of *β*_2_GPI in diabetic patients. Therefore, this study aimed to explore the relationship between the redox states of *β*_2_GPI in diabetics and at different stages of angiopathy.

## 2. Materials and Methods

### 2.1. Ethics Statement

This study was approved by the ethics committee of Metabolic Disease Hospital, Tianjin Medical University, and written informed consents were obtained from all subjects before their enrollment into the study. Assays were performed under blind condition.

### 2.2. Materials and Reagents

N-(3-maleimidylpropionyl) biocytin (MPB) was purchased from Invitrogen (USA). Alkaline phosphatase- (AP-) conjugated anti-mouse IgG was purchased from Boster (China). TRX-1 was purchased from R&D (USA). TRXR (Thioredoxin Reductase) was from Sigma (USA). NADPH was from Calbiochem Merck (USA). Affinity purified murine IgG2 anti-*β*_2_GPI monoclonal antibody (mAb) 4B2E7 and affinity purified rabbit anti-*β*_2_GPI polyclonal antibody were from Department of Immunology, Allergy, and Infectious Diseases and Department of Medicine, University of New South Wales, Sydney, Australia [6, 7]. High-binding 96-well plates were from JETBIOFIL, Guangzhou (China). The human AGEs ELISA Kit was from Cusabio Biotech, Wilmington, DE (USA).

### 2.3. Patients and Their Clinical Information

230 T2DM patients and 80 healthy controls were enrolled from July 2013 to December 2013. Subjects were excluded if they had autoimmune disease [2, 8], acute inflammation, pregnancy or had surgery recently [9–11]. Diabetic ketoacidosis, hyperosmolar hyperglycemia, acute lactic acidosis, and the use of drugs that could change redox state of the body were also excluded from the study [12].

Age, sex, Body Mass Index (BMI), Waist-Hip Ratio (WHR), systolic blood pressure (SBP), diastolic blood pressure (DBP), history of cardiovascular disease and hypertension, diabetic history, and diabetic duration were obtained from the patients' medical records. Fasting blood glucose (FBG), P2BG, HbA1c, platelet (PLT), red blood cell (RBC), hemoglobin, FIB, D-Dimer, blood urea nitrogen (BUN), creatinine (SCR), hepatic function, total cholesterol (CHO), triglyceride (TG), high-density lipoprotein cholesterol (HDL-c), low-density lipoprotein cholesterol (LDL-c), 24 h urine microalbumin (UMA), and 24 h urine total protein (UTP) were all determined by standard clinical biochemical assays. Nerve Conduction Velocity Test (NCVT) was done using Electromyography/Evoked Potentials Equipment (NDI-200P+, Shanghai). Lower limb arteries were tested by Color Doppler Ultrasound (JYQ TCD-2000). The Ocular Fundus Test was performed using an ophthalmoscope (TOP-CON TRC-NW7SF), and skin AGEs were measured by AGEs Reader (The Netherlands). The relations between complications of CAD, artery stenosis, abnormal nerve conduction velocity, diabetic retinopathy, and abnormal urinary albumin excretory rate and *β*_2_GPI were analyzed. CAD was diagnosed based on one or more of the following criteria: electrocardiogram, echocardiogram, and myocardial perfusion imaging showing myocardial ischemia or infarction. Coronary angiogram or CTA revealed one or more main brunches of coronary artery with more than 50% stenosis.

### 2.4. Determination of Reduced *β*_2_GPI and Total *β*_2_GPI in Serum

We adopted double-antibody sandwich ELISA to quantify total *β*_2_GPI in serum samples [2]. Briefly, high-binding 96-well plates were coated overnight at 4°C with rabbit polyclonal anti-human *β*_2_GPI (10 nM). Plates were washed 4 times with PBS-0.1% Tween and then blocked with 2% BSA/PBS-0.1% Tween for 1 hour at room temperature (RT). Following washing, 100 *μ*L of anti-human *β*_2_GPI mouse mAb was added (10 nM) and then 100 *μ*L of the patient sample (diluted 4,000-fold in PBS-0.05% Tween) was coincubated for 1 hour at RT. After washing 4 times, AP-conjugated goat anti-mouse IgG was added (1 : 1,500) and incubated for 1 hour at RT. Five serum samples were randomly mixed to create a standard serum (internal control), which was used to construct an in-house standard curve for every ELISA. The mixture was aliquoted into Eppendorf tubes, snap frozen, and stored at −80°C. The level of total *β*_2_GPI in standard serum was defined as 100%. Samples were read at 450 nm after addition of chromogenic substrate. Samples were assayed in duplicate.

The relative amount of reduced *β*_2_GPI in patient samples was assayed as previously described [2]. MPB (4 mM) was added to 50 *μ*L of patient plasma or serum and incubated for 30 minutes at RT in dark and then the mixture was diluted 50-fold in 20 mM HEPES buffer (pH 7.4) and incubated for another 10 min at RT in dark. Proteins were then acetone precipitated. Protein pellets were resuspended in PBS-0.05% Tween. The samples were diluted 4000-fold and then added, in duplicate, to a streptavidin-coated 96-well plate (100 *μ*L/well), followed by incubation for 90 minutes at RT. Before adding MPB-labeled serum samples, streptavidin-coated plates were washed 3 times with PBS-0.05% Tween and blocked with 2% BSA/PBS-0.1% Tween. After washing 3 times with PBS-0.1% Tween, the murine anti-*β*_2_GPI mAb was added (25 nM) and incubated for 1 hour at RT. After washing 3 times with PBS-0.1% Tween, alkaline phosphatase-conjugated goat anti-mouse IgG (1 : 1,500) was added for 1 hour at RT and samples were read at 405 nm after adding chromogenic substrate. HAS, non-MPB-labeled serum and ox-*β*_2_GPI were used as controls. The pooled in-house standard used for the above-described *β*_2_GPI quantification ELISA was used as an internal control and standard. The proportion of reduced *β*_2_GPI was expressed as a percentage of that observed with the pooled in-house standard, after correction for the total amount of *β*_2_GPI.

### 2.5. Statistical Analyses

The data were presented as mean and standard deviation. Statistical analyses were performed using SPSS20.0. For normal distribution data, we used independent-samples *t*-test and ANOVA. Nonnormal distribution variables were expressed as medians and interquartile ranges (IQRs) and analyzed by rank sum test. Differences in frequency of categorical variables were assessed by the chi-square test. All reported *p* values were two-sided and values of *p* < 0.05 were considered statistically significant.

## 3. Results

### 3.1. Different Stages of Diabetic Complications in the Patients

In this study, 90 patients did not have CAD (non-CAD group), 56 patients suffered from asymptomatic myocardial ischemia or stable angina (mild-CAD group), and 84 patients had unstable angina or a demonstrated Myocardial Infarction (severe-CAD group). According to Doppler ultrasonography, 108 patients were free of artery stenosis of lower limbs (non-A-stenosis group), 63 patients had less than 50% artery stenosis (mild-A-stenosis group), and 59 patients had more than 50% artery stenosis (severe-A-stenosis group). 47 patients had normal nerve conduction velocity (NCVN group), 99 patients had slow nerve conduction velocity, as judged by the slowing of nerve conduction velocity less than 30% of the control (mild-NCVS group), and 84 patients had slow NCVS by more than 30% of the controls (severe-NCVS group). According to the Diabetic Retinopathy Disease Severity Scale, 138 patients did not have diabetic retinopathy (non-DR group), 56 patients suffered from mild-moderate nonproliferative diabetic retinopathy (mild-DR group), and 36 patients had severe NPDR-proliferative diabetic retinopathy (severe-DR group). According to Mogensen staging criteria, 163 patients had normal urinary albumin excretory rate (DN < III group), 44 patients had stage III-DN (DN-III group), and 23 patients had stage IV-V-DN (DN-IV-V group). Demographic and clinical details of the study groups are summarized in supplemental Tables (see Supplementary Material available online at http://dx.doi.org/10.1155/2016/8246839).

### 3.2. The Proportion of Reduced *β*_2_GPI Is Decreased in T2DM

**Figure 1:**
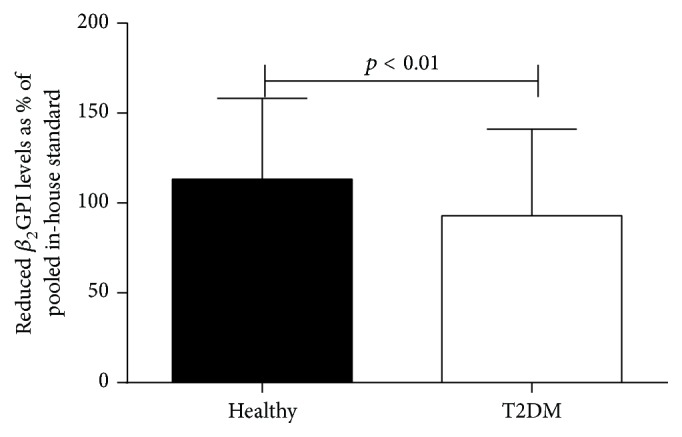
The proportion of reduced *β*_2_GPI decreased in T2DM.

The proportion of reduced *β*_2_GPI was significantly lower in T2DM group (92.98% ± 48.05%) than in healthy control group (113.27% ± 44.99%) (*p* < 0.01) (Figure 1).

### 3.3. Reduced *β*_2_GPI Was Elevated at Early Stage of Diabetic Macroangiopathy but Decreased at the Late Stage

**Figure 2:**
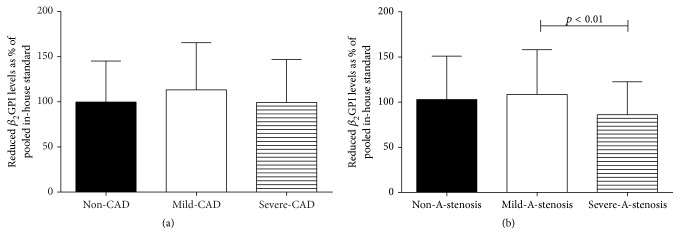
*β*_2_GPI and macroangiopathy.

Given that the redox state of *β*_2_GPI in T2DM was different from healthy controls, we kept asking whether this level was the same in patients with various degrees of angiopathy. We found that there was a trend that the mild-CAD group had higher proportion of reduced *β*_2_GPI (113.25% ± 45.42%) than non-CAD (99.76% ± 45.42%) and severe-CAD groups (99.26% ± 47.44%) (Figure 2(a)), however without significant difference (*p* > 0.05). The mild-A-stenosis group had higher proportion of reduced *β*_2_GPI (108.63% ± 49.45%) than severe-A-stenosis group (86.10% ± 36.42%) (*p* < 0.05) and non-A-stenosis group (102.96% ± 47.96%) (*p* > 0.05) (Figure 2(b)).

**Figure 3:**
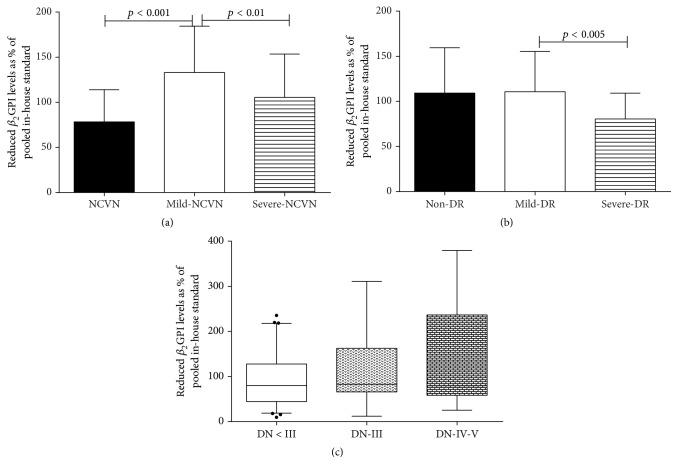
The levels of *β*_2_GPI in different NCVS (a), DR (b), and DN (c).

The mild-NCVS group had a higher proportion of reduced *β*_2_GPI (133.00% ± 51.44%) than NCVS group (78.78% ± 35.62%) (*p* < 0.001) and severe-NCVS group (105.43% ± 48.02%) (*p* < 0.01) (Figure 3(a)). Mild-DR group had higher proportion of reduced *β*_2_GPI (110.67% ± 44.58%) than severe-DR group (80.55% ± 28.50%) (*p* < 0.01) and the proportion of reduced *β*_2_GPI in non-DR group (109.20% ± 50.17%) was almost equal to that in mild-DR group (*p* > 0.05) (Figure 3(b)). For DN, the proportion of reduced *β*_2_GPI showed no difference between any two groups, as shown in Figure 3(c). However, there was a trend that DN at I or II status had a higher proportion of reduced *β*_2_GPI [82.89% (66.05–162.53)] than DN-III group [79.93% (44.44–127.57)] and DN-IV and V group [79.24% (58.28–236.42)] (*p* > 0.05). Additionally, the proportion of reduced *β*_2_GPI was in positive correlation with 24 h UMA (*r* = 0.138) and 24 h UTP (*r* = 0.134) (*p* < 0.05).

### 3.4. Correlation Analysis

**Figure 4:**
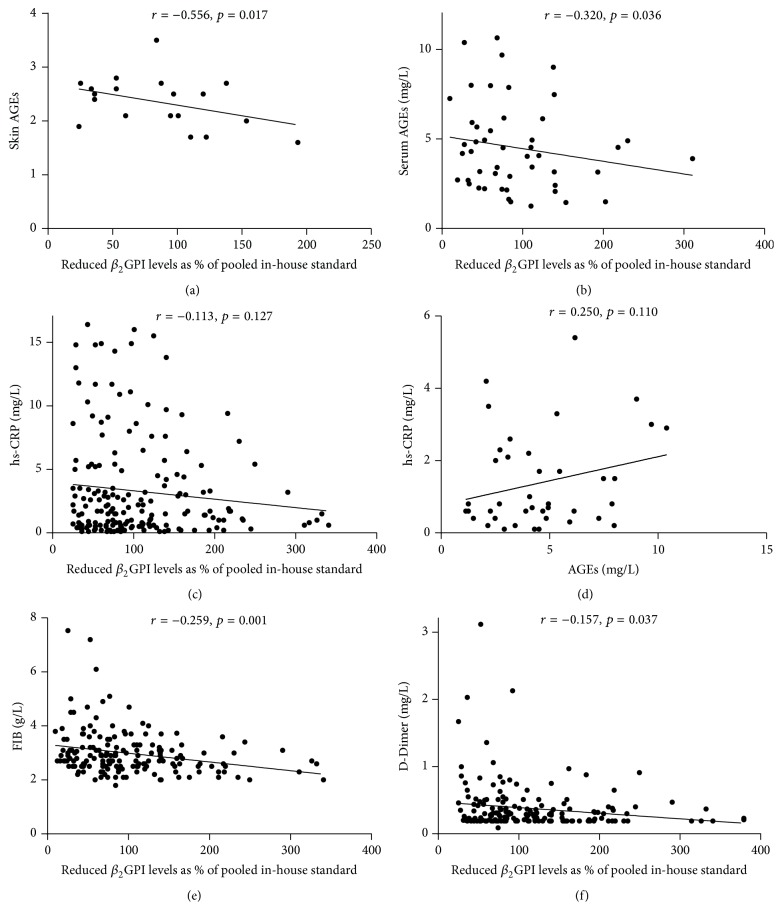
Correlation analysis. Correlation of *β*_2_GPI with skin AGES (a) or with serum AGEs (b). Correlation of *β*_2_GPI with hs-CRP (c). Correlation of hs-CRP with AGEs (d). Correlation of *β*_2_GPI with FIB (e) and D-Dimer (f).

The proportion of reduced *β*_2_GPI was in negative correlation with skin AGEs (*r* = −0.556, *p* < 0.05) (Figure 4(a)), serum AGEs (*r* = −0.320, *p* < 0.05) (Figure 4(b)), hs-CRP (*r* = −0.113, *p* > 0.05) (Figure 4(c)), FIB (*r* = −0.259, *p* < 0.05) (Figure 4(d)), and D-Dimer (*r* = −0.157, *p* < 0.05) (Figure 4(e)). Serum hs-CRP was in negative correlation with serum AGEs (*r* = 0.250, *p* > 0.05) (Figure 4(f)).

## 4. Discussion

### 4.1. Type 2 Diabetes Mellitus and Redox State of *β*_2_GPI

The conformation and function of reduced *β*_2_GPI are quite different from those of the oxidized form [13, 14]. Therefore, ROS is likely to play a direct role in the regulation of *β*_2_GPI status. APS, which is characterized by increased oxidative stress and vascular thrombosis, has elevated *β*_2_GPI and a decreased proportion of reduced *β*_2_GPI [2]. Similarly, diabetics are in a high oxidative stress state due to massive ROS production caused by increased AGEs, the polyol pathway, and other factors. Indeed, our study showed that T2DM patients had significantly lower proportion of reduced *β*_2_GPI. Serum AGEs were in positive correlation with hs-CRP which is an important inflammatory marker and one of the strongest independent predictors of cardiovascular disease, and the proportion of reduced *β*_2_GPI was in negative correlation with hs-CRP, serum and skin AGEs, D-Dimer, and FIB.

### 4.2. Reduced *β*_2_GPI and Diabetes Macroangiopathy

Atherosclerosis (AS) is a complex chronic disease caused by various factors. *β*_2_GPI plasma concentrations are strongly associated to vascular disease in type 2 diabetic patients and have been proposed as a clinical marker of cardiovascular risk [15]. *β*_2_GPI/CRP, oxLDL/*β*_2_GPI, and LP(a)/*β*_2_GPI complexes in serum can accelerate progress of atherosclerosis [16, 17]. *β*_2_GPI can also induce a cellular immune response in a subpopulation of patients with carotid atherosclerosis thus contributing to the inflammatory responses involved in carotid atherosclerotic disease [18]. The T-cell recognition site of *β*_2_GPI is around Cys288-Cys326 disulfide bond and the redox state of this could affect cellular immune processes mediated by *β*_2_GPI. Thus, it can be inferred that *β*_2_GPI in its oxidized form has the potential to be one of the significant pathogenic factors of atherosclerosis.

Reduced *β*_2_GPI can inhibit the formation of foam cells, reduce macrophage apoptosis [5], and protect endothelial cells from oxidative stress-induced cell injury [4]. Our results implicated that the change of redox state of *β*_2_GPI would affect its functions. Reduced *β*_2_GPI could be a protective factor from atherosclerosis, while the oxidized *β*_2_GPI could accelerate the disease. In the present study, we have found that the proportion of reduced *β*_2_GPI appeared a rising trend in the early stage of diabetes macroangiopathy due to the compensatory mechanism and showed a decreasing trend in the late stage of CAD and arterial stenosis, although it was not statistically different. More samples are still needed to clarify the difference.

### 4.3. Reduced *β*_2_GPI and Diabetes Microangiopathy

*β*_2_GPI combines with the A1 domain of vWF, subsequently inhibiting vWF mediated adhesion and aggregation of platelets. Obviously, *β*_2_GPI has the anticoagulant effect by clearing vWF. In addition, anti-*β*_2_GPI autoantibodies can eliminate this effect of *β*_2_GPI [19, 20]. Accordingly, Passam et al. found that reduced *β*_2_GPI had greater affinity with vWF* in vitro* [21].

*β*_2_GPI has the effect to inhibit angiogenesis [22]. *β*_2_GPI DN I-IV can inhibit proliferation, migration of RF/6A cell, and the function of lumen formation induced by AGEs. AGEs upregulate the expression of vascular endothelial growth factor (VEGF) and VEGF receptor. Reduced *β*_2_GPI functions to inhibit retinal angiogenesis by downregulating the expression of VEGF, VEGFR-1, and VEGFR-2 and inhibiting phosphorylation of ERK1/2 and Akt through Ras/Raf/MEK/ERK and PI3K/Akt/Gsk3*β* pathways.

In our previous study, reduced *β*_2_GPI treated STZ-BALB/c mice had a lower urinary albumin excretion rate (UAER) and a less pronounced pathological damage of kidney compared with control mice [16]. Western blots showed that reduced *β*_2_GPI treated group had less expression of phosphorylated p38MAPK, TGF-*β*1, and type IV collagen. Reduced *β*_2_GPI eliminates vWF, inhibits retinal angiogenesis, and inhibits glomerular fibrosis, thereby inhibiting the development of DR and DN. In our study, the proportion of reduced *β*_2_GPI increased in the early stage of diabetes microangiopathy and then decreased with aggravating of diabetes microangiopathy. Therefore, reduced *β*_2_GPI may be a protective factor in diabetes microangiopathy.

The redox state of *β*_2_GPI changed in T2DM patients. The proportion of reduced *β*_2_GPI increased in the early stage of angiopathy possibly as a compensatory mechanism and then decreased as angiopathy aggravated. This may implicate that reduced *β*_2_GPI is a protective factor in diabetes angiopathy. Testing the amount and proportion of reduced B_2_GPI periodically in “at-risk” patients may offer the potential to better predict the occurrence and development of diabetic angiopathy.

## Supplementary Material

Table 1. Demographic and clinical characteristics of the groups studied. Data are presented as means ± standard deviation, median (interquartile range) or number (%). In table 1-1 and table 1-2, the demographic and clinical characteristics between control and T2DM were compared. As shown in table 1-1 and table 1-2, following parameters had significant difference: BMI, WHR, SBP, FBG, P2BG, HbA1c, TG, TC, HDL-c, LDL-c, VLDL-c, Hb, PLT, SCr, BUN, SUA, hs-CRP, D-Dimer, FIB, A/G, 24 h UTP, 24 h UMA. Following parameters had significant difference among non-A-stenosis, middle-A-stenosis and severe-A-stenosis: Age, Sex, WHR, diabetic duration, smoking, drinking, SBP, FBG, TG, TC, SCr, BUN, SUA, hs-CRP, D-Dimer, FIB, Albumin, 24 h UTP, 24 h UMA. As shown in table 1-3, 1-4, following parameters had significant difference among non-NCVS, NCVS-mild and NCVS-severe: Sex, BMI, diabetic duration, drinking, HbA1c, HDL-c, SCr, BUN, SUA, hs-CRP, D-Dimer. Following parameters had significant difference among non-DR, middle-DR and severe-DR: WHR, smoking, drinking, SBP, HbA1c, HDL-c, Hb, SCr, SUA, FIB, TBIL, DBIL, 24 h UTP, 24 h UMA. Following parameters had significant difference among DN<III, DN-III and DN-IV-V: Sex, BMI, WHR, smoking, drinking, SBP, Hb, SCr, BUN, SUA, D-Dimer, FIB, Albumin, A/G, TBIL, DBIL, γ-GGT
