# Supplementary material for: Redox Status of β 2GPI in Different Stages of Diabetic Angiopathy
Source: Dis Markers. 2016 Oct 13;2016:8246839. doi: 10.1155/2016/8246839 (PMC5081428; doi:10.1155/2016/8246839)

**List of Supporting Information**

**Supplemental tables** Demographic and clinical characteristics of the groups studied.


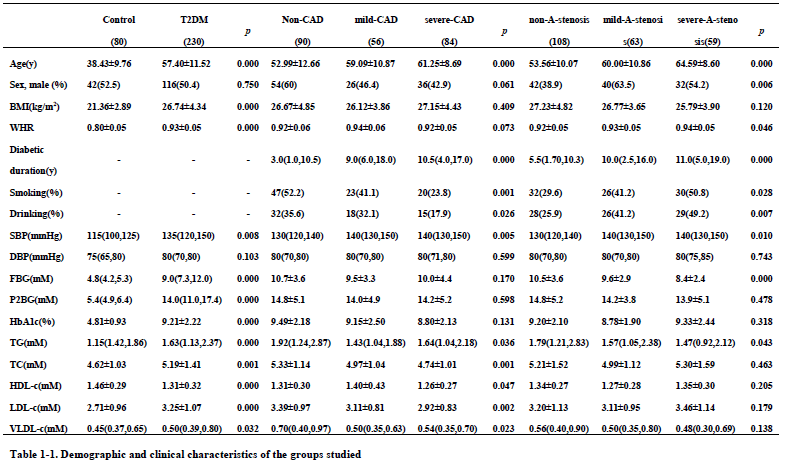


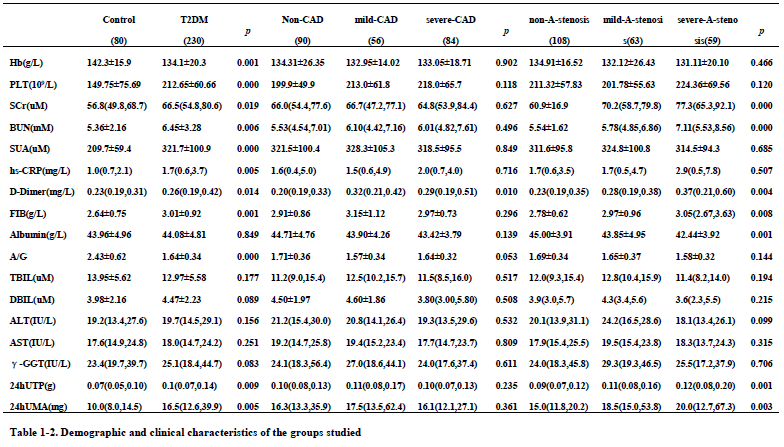


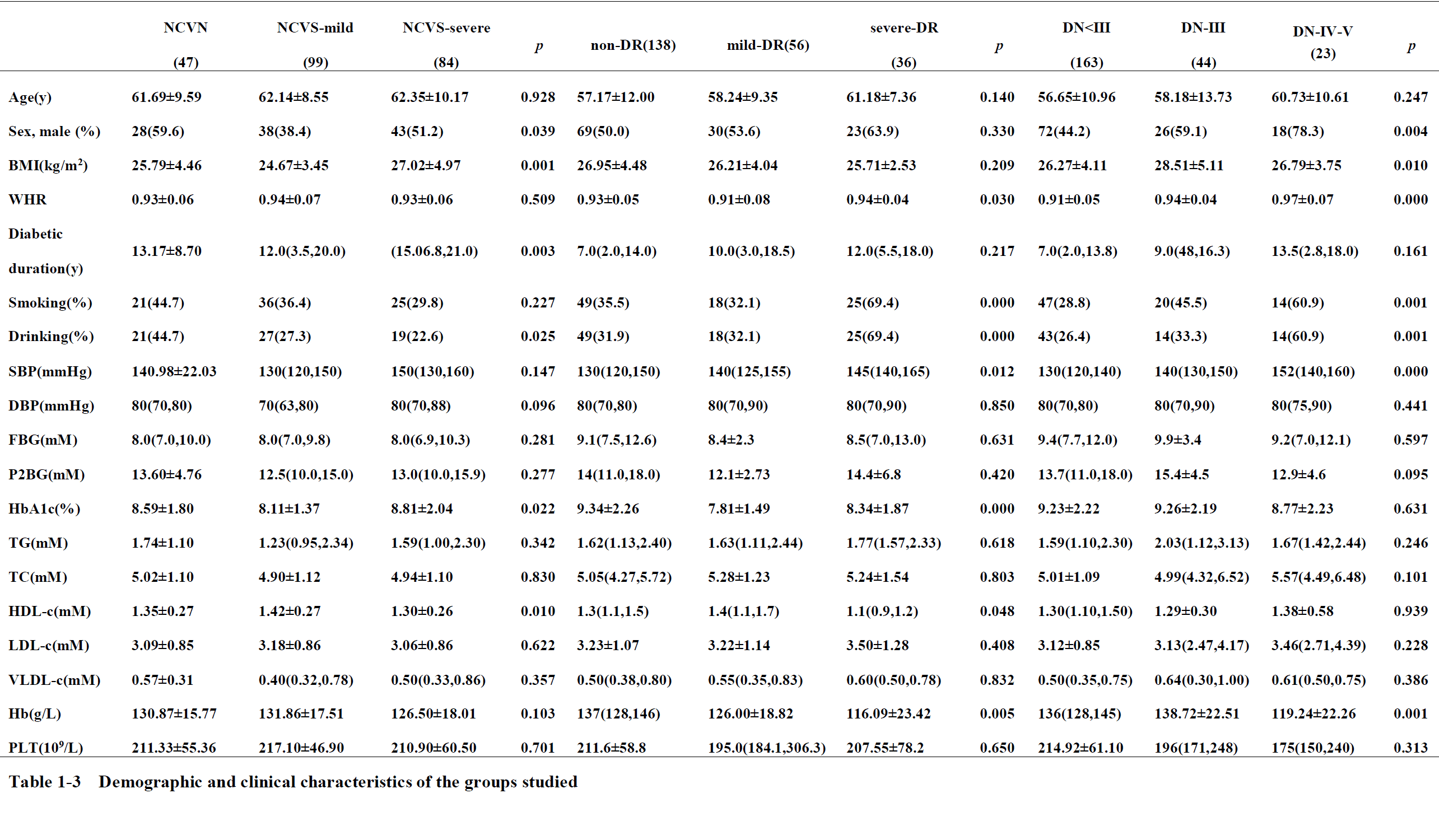


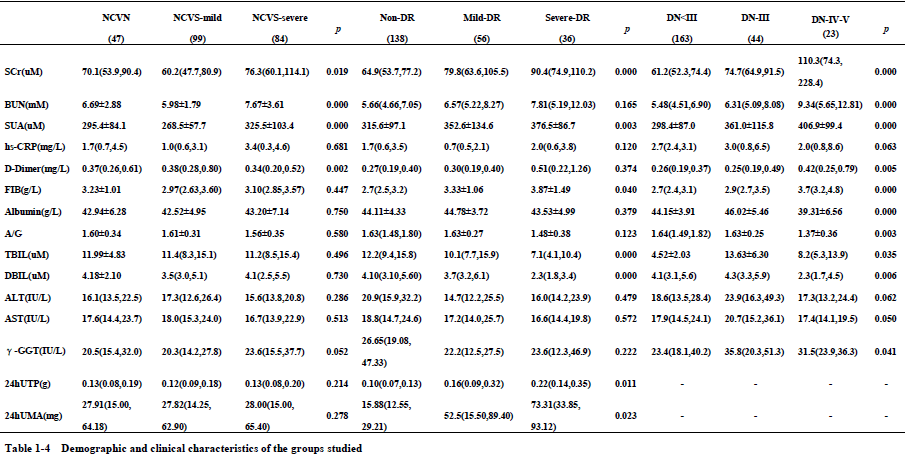

Supplement: Supplementary file 1 — Table 1. Demographic and clinical characteristics of the groups studied. Data are presented as means ± standard deviation, median (interquartile range) or number (%). In table 1-1 and table 1-2, the demographic and clinical characteristics between control and T2DM were compared. As shown in table 1-1 and table 1-2, following parameters had significant difference: BMI, WHR, SBP, FBG, P2BG, HbA1c, TG, TC, HDL-c, LDL-c, VLDL-c, Hb, PLT, SCr, BUN, SUA, hs-CRP, D-Dimer, FIB, A/G, 24 h UTP, 24 h UMA. Following parameters had significant difference among non-A-stenosis, middle-A-stenosis and severe-A-stenosis: Age, Sex, WHR, diabetic duration, smoking, drinking, SBP, FBG, TG, TC, SCr, BUN, SUA, hs-CRP, D-Dimer, FIB, Albumin, 24 h UTP, 24 h UMA. As shown in table 1-3, 1-4, following parameters had significant difference among non-NCVS, NCVS-mild and NCVS-severe: Sex, BMI, diabetic duration, drinking, HbA1c, HDL-c, SCr, BUN, SUA, hs-CRP, D-Dimer. Following parameters had significant difference among non-DR, middle-DR and severe-DR: WHR, smoking, drinking, SBP, HbA1c, HDL-c, Hb, SCr, SUA, FIB, TBIL, DBIL, 24 h UTP, 24 h UMA. Following parameters had significant difference among DN<III, DN-III and DN-IV-V: Sex, BMI, WHR, smoking, drinking, SBP, Hb, SCr, BUN, SUA, D-Dimer, FIB, Albumin, A/G, TBIL, DBIL, γ-GGT [file 8246839.f1.docx]
